# Antimicrobial activity of cell free supernatants from probiotics inhibits against pathogenic bacteria isolated from fresh boar semen

**DOI:** 10.1038/s41598-023-33062-w

**Published:** 2023-04-12

**Authors:** Krittika Keeratikunakorn, Thotsapol Kaewchomphunuch, Kampon Kaeoket, Natharin Ngamwongsatit

**Affiliations:** 1 Department of Clinical Sciences and Public Health, Faculty of Veterinary Science, Mahidol University, 999 Phuttamonthon 4 Rd., Salaya, Phuttamonthon, Nakhon Pathom, 73170 Thailand; 2 Laboratory of Bacteria, Veterinary Diagnostic Center, Faculty of Veterinary Science, Mahidol University, 999 Phuttamonthon 4 Rd., Salaya, Phuttamonthon, Nakhon Pathom, 73170 Thailand

**Keywords:** Microbiology, Antimicrobials, Bacteria

## Abstract

The use of antibiotics with semen extender appears to be a practical solution to minimise bacterial growth in fresh boar semen preservation. Unfortunately, the excessive use of antibiotics promotes antimicrobial resistance (AMR). This becomes a worldwide concern due to the antimicrobial resistance genes transmitted to animals, environment, and humans. Probiotics are one of the alternative methods to reduce antibiotic use. They could inhibit pathogenic bacteria by producing antimicrobial substances in cell free supernatants (CFS). Nevertheless, there is no comprehensive study undertaken on inhibitory activity against pathogenic bacteria isolated from boar semen origin. Our study investigated the efficacy of CFS produced from selected probiotics: *Bacillus* spp., *Enterococcus* spp., *Weissella* spp., *Lactobacillus* spp., and *Pediococcus* spp. inhibiting pathogenic bacteria isolated from fresh boar semen. Besides, the semen-origin pathogenic bacteria are subjected to identification, antimicrobial resistance genes detection, and antibiotic susceptibility test (AST). *Pseudomonas aeruginosa*, *Escherichia coli*, and *Proteus mirabilis* are the most common pathogens identified in boar semen with resistance to numerous antibiotics used in pig industry. The CFS with its antimicrobial peptides and/or bacteriocin constituent derived from selected probiotics could inhibit the growth of pathogenic bacteria carrying antimicrobial resistance genes (*mcr-3* and *int1* genes). The inhibition zones for *Pseudomonas aeruginosa*, *Escherichia coli*, and *Proteus mirabilis* provided more efficient results in the CFS derived from *Lactobacillus* spp. and *Pediococcus* spp. than those of the CFS produced from *Enterococcus* spp., *Weissella* spp. and *Bacillus* spp., respectively. It is worth noted that as the incubation time increased, the antibacterial activity decreased conversely. Our results on CFS with its antimicrobial peptides and/or bacteriocin constituent inhibits semen-origin pathogenic bacteria guide the direction as a promising alternative method used in the semen extender preservation of the pig industry.

## Introduction

Bacterial contamination in fresh boar semen plays an important role in semen quality. Negative impacts affect fertility rate, embryonic or foetal death, and endometritis in sows/gilts after insemination^1,2^. The clinical appearance of endometritis is commonly observed with vaginal discharge^3,4^ which may occur due to different causes, e.g. hormonal imbalance^5^ or post-ovulatory insemination^6,7^. Although the severity of acute endometritis can be alleviated with antibiotics, acute endometritis can be progressive and turn into chronic endometritis, resulting in significant impacts on reproductive performance^3^.

Preservation of boar semen is a routine process for artificial insemination (AI) in the swine industry^8^. There are numerous advantages of AI, such as transmitted disease prevention, genetic improvement, piglet production, and quality enhancement^1,8^. Although AI can potentially reduce the rate of disease transmission from boar semen, microbial contamination in boar semen is an issue of concern since it also plays a major role in reproductive performance^1^.

Microbial contamination occurs during the process of semen collection and is derived from either animal or non-animal origin^9,10^. In addition, Gram-negative bacteria are the most predominant (more than 80%) from fresh boar semen isolation^11^. The significant impacts of bacterial contamination on semen quality include: (i) sperm motility reduction; (ii) increased sperm agglutination; (iii) acrosomal damage sperm; and (iv) plasma membrane disruption^9,10^. The relationship between boar semen quality and farm production reveal that sperm agglutination due to *Escherichia coli* (*E*. *coli*) contamination can markedly reduce litter size^12^. In practice, numerous antibiotics are mixed into the semen extender with the aims of inhibiting bacterial growth and limiting the negative impacts from the contamination1^3–15^. For instance, gentamicin, neomycin, streptomycin, and other antibiotics are commonly used in boar semen extender^16–18^. In addition, more than one antibiotic can be combined with the boar semen extender, for example gentamicin and florfenicol or gentamicin and polymyxin B combinations have been used^19^. Consequently, the popularity of antibiotics to maintain farm production raises questions about the reasonableness for antibiotics use as well as the concern about antibiotic resistant bacteria^20^.

Colistin (polymyxin E) was discovered in 1947 as a secondary metabolite of *Paenibacillus polymyxa* subsp. *Colistinus*^21,22^. The use of colistin is conserved as a last-line antibiotic for humans in the treatment of serious infection caused by multidrug resistant Gram-negative bacteria^23^. Due to excessive use of colistin, there is an incidence of mobile colistin resistance (*mcr*) genes which is developed with chromosomal mutations and is plasmid-mediated in numerous bacterial species. Recently, the identification of mobilized colistin resistance genes has been reported as *mcr-1* to *mcr-10* and the *mcr-1* gene is the most predominant^23,24^. By considering the mechanisms, the *mcr-1* gene encodes phosphoethanolamine transferase which plays an important role in modification of lipopolysaccharides (LPS) in the outer surface of Gram-negative bacteria by adding phosphoethanolamine (PEA) to lipid A moieties. It then causes a lower affinity of colistin to its primary target^25–27^. The integron-integrase gene is an essential source of gene cassettes with horizontal gene transfer of antibiotic resistance. Consequently, this gene plays a crucial role in the spread and transmission of antibiotic-resistant determinants in resistant bacteria^28^. There are three classes of integron-integrase genes, with the class 1 integron-integrase (*int1*) gene is the most predominant^29,30^.

Some compounds (i.e. antimicrobial peptides and bacteriocins) in probiotics cell free supernatants (CFS) can inhibit the growth of other bacteria^31^. CFS with its antimicrobial peptides and/or bacteriocin constituent derived from probiotics, especially lactic acid bacteria (LAB), exhibit the inhibitory activities against various pathogenic bacteria. Previous studies of the antimicrobial activities of CFS have investigated various pathogens, including *E*. *coli*^32^, *Salmonella* Typhi and *Salmonella* Typhimurium^33,34^, *Listeria monocytogenes*^35^, and *Staphylococcus aureus*^34^. Although many authors have carried out the antimicrobial effects of CFS, there are vast gaps in CFS antimicrobial properties in bacterial isolation on animals. In particular, the pathogenic bacteria carrying antimicrobial resistance genes isolated from fresh boar semen are scarce and not comprehensive. The effect of CSF on inhibition the growth of other bacteria may guide the direction of finding a promising alternative method of using CFS-origin antimicrobial peptides and/or bacteriocin in the semen extender preservation of the pig industry.

Here, our work aims to identify the bacterial species, conduct antibiotic susceptibility testing (AST), and detect antimicrobial resistance genes (*mcr-1* to *mcr-10* and *int1*) isolated from fresh boar semen in Thailand. Furthermore, the study of inhibitory activities of CFS derived from *Bacillus* spp., *Enterococcus* spp., *Weissella* spp., *Lactobacillus* spp., and *Pediococcus* spp. against pathogens isolated from fresh boar semen are also investigated.

## Results

### Bacterial identification

Bacterial from fresh boar semen (n = 10) were successfully identified and confirmed using 16S rRNA gene sequencing. The bacterial identification and percentage of identity were presented in Table 1. The semen sample were identified as 10 bacterial species and classified into three species for Gram-positive bacteria and seven species of Gram-negative bacteria (Table 1). The three major pathogens identified of this study were *P*. *aeruginosa* (5/10; 50%), *E*. *coli* (4/10; 40%), and *P*. *mirabilis* (3/10; 30%). The other Gram-negative bacteria including *Citrobacter koseri*, *Enterobacter hormaechei*, *Providencia stuartii*, and *P*. *alcaligenes* showed one isolate (1/10; 10%). In addition, the Gram-positive bacteria were identified as *Staphylococcus* spp. (*S*. *chromogenes*, *S*. *sciuri*, and *S*. *warneri*).

**Table 1:** Bacterial identification, antimicrobial susceptibility against 10 antimicrobial agents, and antimicrobial resistant genes detection of pathogens isolated from fresh boar semen

Bacterial isolation	Swine farms (location)	Sample ID	MIC (μg/mL)										Antimicrobial resistance genes
			CN	CAZ	CEF	CT	ENR	AMX	AMC	CRO	OTC	SXT	
*Pseudomonas aeruginosa*	Farm A (Chai Nat)	S2NLF	2	4	**32**	2	0.5	**>128**	16	**16**	**32**	**>32**	-
	Farm A (Chai Nat)	S3NLF	**>64**	**>128**	**16**	1	0.25	**128**	8	**8**	**32**	**>32**	-
	Farm A (Chai Nat)	S4NLF2	<0.5	<1	**16**	1	0.125	**>128**	4	**16**	**>32**	**>32**	-
	Farm B (Chon Buri)	S7	<0.5	<1	**16**	4	0.5	**>128**	16	**>128**	**>32**	**>32**	-
	Farm B (Chon Buri)	S8-4	4	<1	**8**	1	0.125	**64**	8	**>128**	**>32**	**16**	*int1*
*Escherichia coli*	Farm A (Chai Nat)	S4LF3	1	<1	0.5	1	0.5	**>128**	<1	**4**	4	**16**	-
	Farm A (Chai Nat)	S5LF3	<0.5	<1	0.5	0.5	0.5	**>128**	<1	**4**	4	**8**	-
	Farm B (Chon Buri)	S7-2LF	**64**	<1	**>32**	2	**8**	**>128**	**128**	**16**	**>32**	**16**	*mcr-3*,* int1*
	Farm B (Chon Buri)	S8-1LF	**64**	8	**8**	1	**8**	**>128**	16	**>128**	2	**32**	-
*Proteus mirabilis*	Farm A (Chai Nat)	S1NLF	1	<1	<0.25	**32**	<0.125	**>128**	<1	**>128**	1	1	*int1*
	Farm A (Chai Nat)	S3	<0.5	<1	<0.25	**16**	0.25	**128**	<1	**128**	1	2	*int1*
	Farm A (Chai Nat)	S4	1	<1	<0.25	**32**	<0.125	**>128**	<1	**>128**	1	2	*int1*
*Citrobacter koseri*	Farm A (Chai Nat)	S1LLF	<0.5	<1	2	1	<0.125	**64**	4	**>128**	1	2	*int1*
*Enterobacter hormaechei*	Farm B (Chon Buri)	S8-6LFmu	<0.5	<1	1	2	0.5	**>128**	8	**>128**	**>32**	**>32**	*int1*
*Providencia stuartii*	Farm A (Chai Nat)	S4NLF1	2	<1	0.5	**>32**	<0.125	**128**	<1	**>128**	4	**32**	-
*Pseudomonas alcaligenes*	Farm B (Chon Buri)	S6-4NLF	<0.5	<1	4	0.5	<0.125	16	8	<1	2	**8**	-
*Staphylococcus chromogenes*	Farm C (Chachoengsao)	S10	8	**32**	4	**32**	0.5	4	16	**>128**	1	0.5	-
*Staphylococcus sciuri*	Farm B (Chon Buri)	S7-3W	4	**64**	**8**	4	0.25	<1	**64**	**>128**	**>32**	0.5	-
*Staphylococcus warneri*	Farm C (Chachoengsao)	S9-1	<0.5	8	<0.25	**16**	0.125	<1	<1	<1	1	<0.25	-

*CN* Gentamicin, *CAZ* Ceftazidime, *CEF* Ceftiofur, *CT* Colistin, *ENR* Enrofloxacin, *AMX* Amoxicillin, *AMC* Amoxicillin trihydrate: Potassium clavulanate (4:1), *CRO* Ceftriaxone, *OTC* Oxytetracycline, *SXT* Trimethoprim: sulfamethoxazole (1:19)

Bold indicate the resistant zone

Chai Nat province is in central region of Thailand. Chonburi and Chachoengsao provinces are in eastern region of Thailand.

### Antibiotic susceptibility test (AST*)*

Bacterial identification from boar semen were tested for antimicrobial susceptibility with 10 selected antibiotics. Most Gram-negative bacterial isolates were resistant to amoxicillin and ceftriaxone except for *P*. *alcaligenes* (Table 1). All *P*. *aeruginosa* isolates were resistant to ceftiofur (MIC > 8 μg/mL), amoxicillin (MIC > 32 μg/mL), ceftriaxone (MIC > 4 μg/mL), oxytetracycline (MIC > 6 μg/mL), and trimethoprim:sulfamethoxazole (1:19) (MIC > 8 μg/mL) (Table 1). Meanwhile *P*. *aeruginosa* was susceptible to colistin (MIC < 4 μg/mL), enrofloxacin (MIC < 2 μg/mL), and amoxicillin trihydrate:potassium clavulanate (4:1) (MIC < 32 μg/mL) (Table 1).

Similarly, all *E*. *coli* strains were resistant to amoxicillin (MIC > 32 μg/mL), ceftriaxone (MIC > 4 μg/mL), and trimethoprim:sulfamethoxazole (1:19) (MIC > 8 μg/mL), but susceptible to ceftazidime and colistin (MIC < 4 μg/mL) (Table 1). Furthermore, the 50% of *E*. *coli* isolates was susceptible to gentamicin (MIC < 16 μg/mL), ceftiofur (MIC < 8 μg/mL) as well as enrofloxacin (MIC < 2 μg/mL) (Table 1). *P*. *mirabilis* was resistant to colistin (MIC > 4 μg/mL), amoxicillin (MIC > 32 μg/mL), and ceftriaxone (100 %) (MIC > 4 μg/mL). All Gram-positive bacteria isolated from boar semen were susceptible to gentamicin (MIC < 16 μg/mL), enrofloxacin (MIC < 2 μg/mL), amoxicillin (MIC < 32 μg/mL), and trimethoprim:sulfamethoxazole (1:19) (MIC < 4 μg/mL) (100 %) (Table 1).

### Detection of *mcr* and *int1* genes

The detection of *mcr-1* to *mcr-10* and *int1* in all pathogens isolated from fresh boar semen were performed using multiplex PCR and showed in Table 1. The *int 1* gene positive was detected in 3 strains of *P*. *mirabilis*, 1 isolate of each *P*. *aeruginosa*, *E*. *coli*, *C*.* koseri* and *E*. *hormaechei* from both Farm A and B. Most pathogens in this study were not contained *mcr-1* to *mcr-10* genes except only one *E*. *coli* isolate from Farm B showed positive to *mcr-3*. This *E*. *coli* strain also exhibited the *int 1* positive and was classified as multidrug resistant according to MIC result. All pathogens which are carried either *int 1* or *mcr-3* were classified as multidrug resistant strains based on the MIC result as shown in Table 1.

### Cell free supernatants (CFS) against pathogens from boar semen

**Figure 1:**
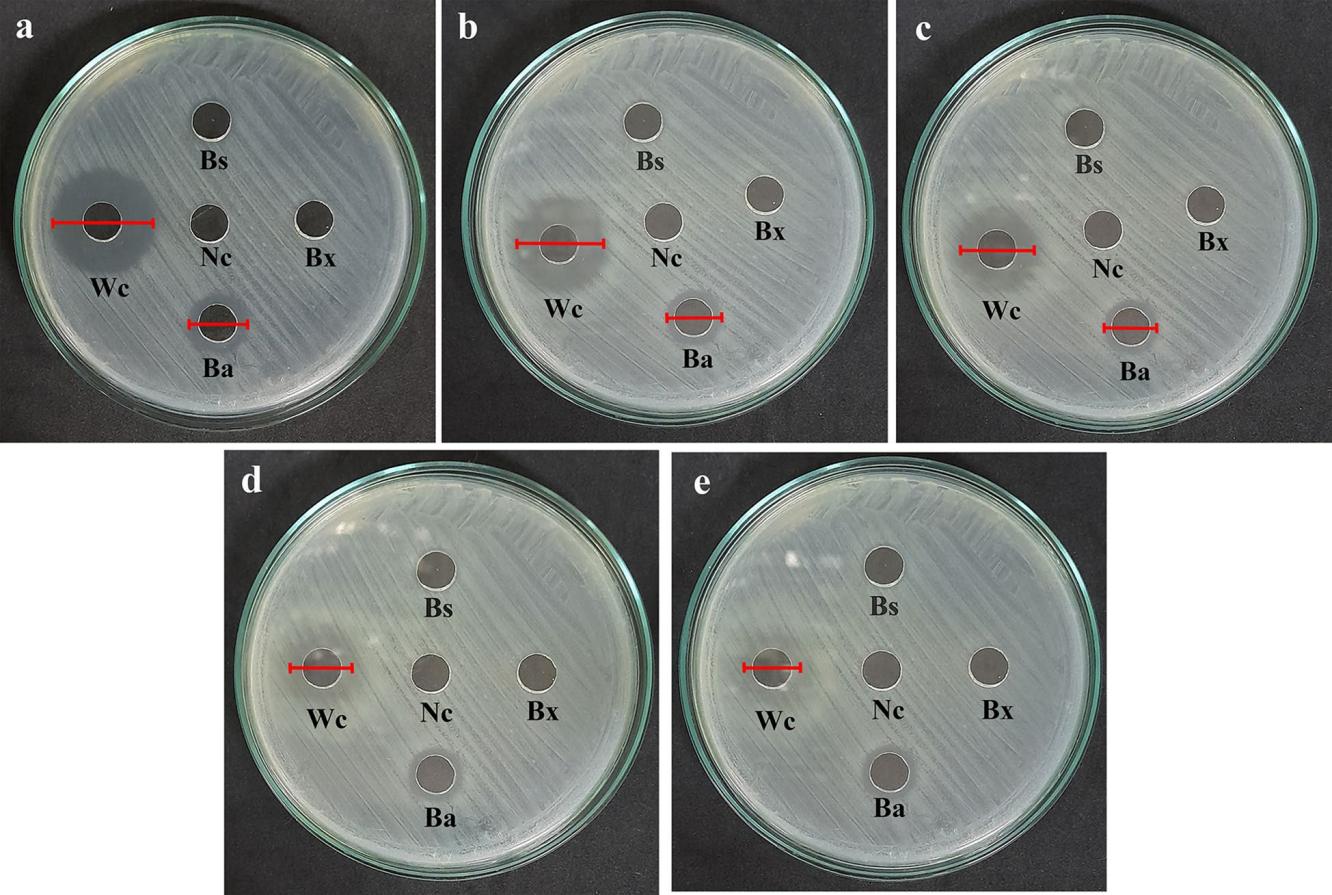
The figure shows the diameter of the inhibition zone of CFS from probiotic bacteria against *Escherichia coli* at 8 h (**a**), 10 h (**b**), 12 h (**c**), 14 h (**d**) and 16 h (**e**) incubation time. The diameter of inhibition zone decreased with increasing incubation time. *Note Ba Bacillus amyloliquefaciens*, *Bs Bacillus subtilis, Bx Bacillus axarquiensis, Nc* Negative control, *Wc Weissella confusa.*

The three selected pathogens from boar semen (*P*. *aeruginosa*, *E*. *coli*, and *P*. *mirabilis*) were conducted to find out the inhibitory effect of CFS from probiotics. The CFS was collected from eight interesting probiotics and their characteristics were declared in Table 2. The antimicrobial activities of CFS were determined as inhibition zone by performing agar well diffusion assay (Fig. 1).

**Table 2:** Characteristics of CFS from selected probiotics.

Probiotics	ID	Source	pH of CFS
*Bacillus amyloliquefaciens*	PB-19-3ML-1	VSMU culture stock	6.0
*Bacillus axarquiensis*	PB-17-3MLS	VSMU culture stock	6.0
*Bacillus subtilis*	KKS 1	VSMU culture stock	6.0
*Enterococcus faecium*	NN28-1M	VSMU culture stock	5.0
*Lactobacillus plantarum*	NN31-5B	VSMU culture stock	4.0
*Pediococcus acidilactici*	NN82-7M	VSMU culture stock	4.0
*Pediococcus pentosaceus*	NN115-6M	VSMU culture stock	4.0
*Weissella confusa*	NN45-2M	VSMU culture stock	5.0

**Figure 2:**
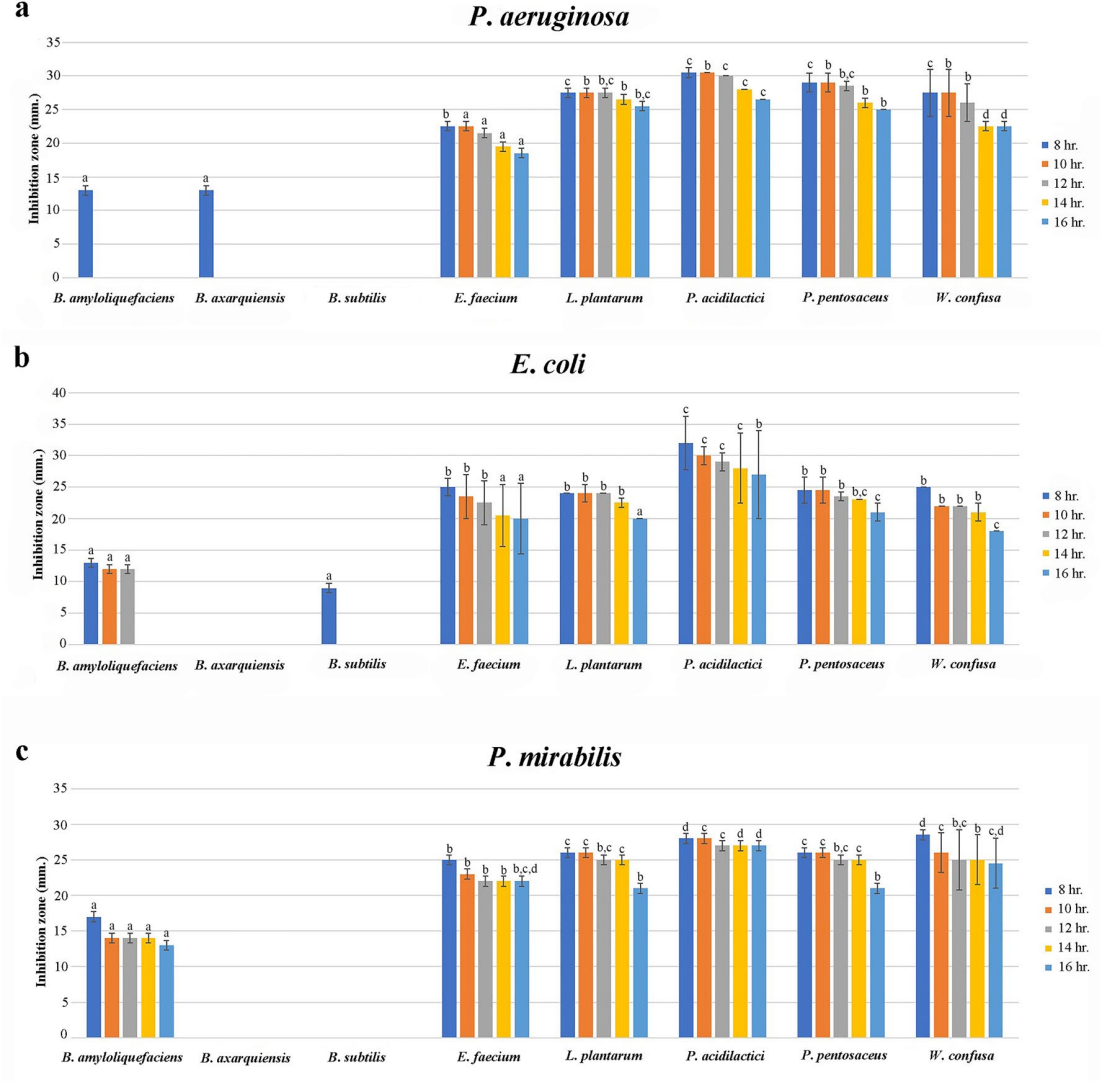
The antimicrobial activity at different time of CFS of probiotic against *Pseudomonas aeruginosa* (**a**), *Escherichia coli* (**b**) and *Proteus mirabilis* (**c**) isolated from boar semen.*Note* ^a,b,c,d^Significant difference between the CFS from probiotics at the same of incubation time (*p* value < 0.05).

The inhibitory activity of CFS against *P*. *aeruginosa* could be initially noticed at 8 h incubation except for the CFS from *B*. *subtilis* (Fig. 2a). At 10 h of incubation, there were no longer inhibitory effects of CFS from *B*. *axarquiensis* and *B*. *amyloliquefaciens*. On the contrary, the CFS produced from *E*. *faecium*, *L*. *plantarum*, *P*. *acidilactici*, *P*. *pentosaceus*, and *W*. *confusa* could remain the inhibitory effect with the presence of similar diameter of inhibition zone at 8, 10, and 12 h incubation. In addition, the inhibitory effect since 12 h incubation was declined in tendency. Interestingly, after 8 hours after incubation, comparing the inhibition zone of CFS from *P*. *acidilactici* and *L*. *plantarum* against *P*. *aeruginosa,* it was found that CSF from *P*. *acidilactici* showed a larger inhibition zone (26.50 to 30.50 mm) than CFS from *L*. *plantarum* (25.50 to 27.50 mm) (*p* value < 0.05).

The interesting probiotics could produce CFS with inhibitory effects against *E*. *coli* except for CFS derived from *B*. *axarquiensis* (Fig. 2b). In details, the CFS produced from *B*. *amyloliquefaciens* and *B*. *subtilis* could inhibit *E*. *coli* in short period of time, 8-10 h and 8 h respectively. On the contrary, the CFS produced from *E*. *faecium*, *L*. *plantarum*, *P*. *pentosaceus*, and *W*. *confusa* thoroughly expressed the inhibitory effects from 8-16 h incubation time. Interestingly, it was apparent that CFS from *P*. *acidilactici* exhibited the largest inhibition zone (27.00 to 32.00 mm) against *E*. *coli.* Furthermore, the inhibitory effects of CFS produced from probiotics against *E*. *coli* shared the same tendency to *P*. *aeruginosa*.

Finally, the CFS produced by *B*. *axarquiensis* and *B*. *subtilis* could not show the inhibitory activities against *P*. *mirabilis* at 8-16 h incubation time (Fig. 2c). At 8-10 h incubation time, the diameter of inhibition zone expressed from *L*. *plantarum*, *P*. *acidilactici*, *P*. *pentosaceus*, and *W*. *confusa* were statistic equivalently. Interestingly, the CFS produced by *P*. *acidilactici* still exhibited the largest diameter of inhibition zone (27.00 to 28.00 mm) thorough the incubation period. In contrary, the CFS from *B*. *amyloliquefaciens* were presented inhibition activity with a smallest inhibition zone (13.00 to 17.00 mm).

Ultimately, it was apparent that the inhibitory activities of probiotics derived CFS shared the same tendency against *P*. *aeruginosa*, *E*. *coli*, and *P*. *mirabilis*. In other words, the inhibitory activities became less effective from the decline of inhibition zone when it had continued for a long period of incubation time.

## Discussion

It was apparent that the bacteria in fresh boar semen could be identified as both Gram-negative and Gram-positive bacteria. *P*. *aeruginosa*, *E*. *coli*, and *P*. *mirabilis* are the predominant bacteria in this study. The results of this survey are found to be similar to a previous survey undertaken in Brazil^38^.In addition, *P*. *aeruginosa* and *E*. *coli* caused negative effects on boar spermatozoa, whether to induce sperm agglutination or decrease sperm motility^10,38^. That effect could be limited by using gentamicin antibiotics in semen extender^10^. The antimicrobial susceptibility test revealed that *P*. *aeruginosa* and *E*. *coli* show susceptibility rates to gentamicin 25% and 50%, respectively, while *P*. *mirabilis* was susceptible to gentamicin for 100%. Additionally, it was observed that sows with endometritis had higher rates of the antimicrobial resistant bacteria. These findings should be of concern since endometritis can be transmitted by bacterial contamination in boar semen^1,37^. In their study, Burch and Sperling^40^ found that 41% of endometritis sows were caused by a single bacterial infection, 72.3% of which were identified as *E*. *coli.* From the result, *E*. *coli* was the most resistant to common antibiotics including amoxicillin and tetracycline, which are used in pig farms and boar semen extender.

The antimicrobial susceptibility test from fresh boar semen revealed that the majority of Gram-negative bacteria were resistant to antibiotic, whereas the Gram-positive bacteria were less antimicrobial resistance. The antibiotic resistance ratio from fresh boar semen was similar to a study undertaken in Italy^41^, while a study in Romania found 56.52% of Gram-negative boar semen bacterial isolation were resistant to gentamicin^42^. This study discovered a high rate of antibiotic drug resistance which increases concerns about the problems caused by the use of antibiotics in agriculture, while the use of antibiotics with semen extender is recommended to protect the spermatozoa^14^. The discovery of novel antimicrobial compounds such as antimicrobial peptides and/or bacteriocin derived from CSF to replace conventional antibiotics is an interesting issue to conduct in the future. It has been shown that the alternative methods were studied to find a feasible way to reduce the use of antibiotics in boar semen extender, including: (i) bacterial removal by a physical method using single-layer centrifugation^43^; (ii) antimicrobial peptides (AMPs) or short antimicrobial lipopeptides^44^; (iii) other substances, such as lysozyme and kojic acid^45,46^; and (iv) semen storage at low temperature conditions (5 °C) without antibiotic supplement^47^. These methods have advantages and disadvantages, such as loss of spermatozoa from the physical method^43^ or no broad-spectrum activity with kojic acid^46^. Moreover, each method has strengths and weaknesses in terms of antimicrobial activity and the effect on semen quality.

Although studies on bacterial contamination and antibiotic resistant bacteria from boar semen are available, no report of antimicrobial resistance genes, particular *mcr-3* gene was found. One boar semen sample was detected for *mcr-3*. By considering the relationship between *mcr-3* detection and colistin resistance, it was found that the sample was susceptible to colistin. The emergence of this incident resembles the study of *mcr-1*, *mcr-4*, and *mcr-5* presented by García *et al*.^48^ as well as *mcr-1* to *mcr-10* by Nguyet *et al*.^49^. These studies utilised five samples of *mcr* genes positive (one sample from *mcr-1* and four samples from *mcr-4*) which were found to be susceptible to colistin^48^. The positive *mcr* gene without colistin resistant spectacle was possible because bacteria were a carrier of an inactive form of the *mcr* gene^48^. Meanwhile the result of four negative *mcr* genes were found to be colistin resistant by the MIC test (three samples from *P*. *mirabilis* and one sample from *P*. *stuartii*). The colistin resistant with the negative *mcr* gene corresponded to a previous study of *E*. *coli*^49^. The colistin resistance was supposed to have another mechanism that does not depend on *mcr* genes. Consequently, the higher incidence of *mcr* genes in livestock animals significantly increased the risk of *mcr* genes being transmitted to humans. In detail, the *mcr* genes could be transmitted to humans via foodborne, zoonotic, and vector-borne routes. Nevertheless, the incidence of the *mcr* gene in humans is higher than the incidence in animals^50^.

The prevalence of the *int1* gene has been studied at different stages of the pig production system. The results indicate that there is a high-rate detection of the *int1* gene in sows and piglets by conducting a rectal swab. Nevertheless, detection of the gene in boars has not been studied^51^. The present study successfully detected the *int1* gene from boars. In addition, the *int1* gene was detected from *P*. *mirabilis*, *P*. *aeruginosa*, *E*. *coli*, *C*.* koseri*, and *E*. *hormaechei*. The *int1* gene has been detected in Gram-negative bacteria and resulted in various antimicrobial resistances, such as β-lactam, sulfonamide, and aminoglycoside^28,52^. According to the related literature, the *int1* gene was not only detected from Gram-negative bacteria but also from Gram-positive bacteria including *Staphylococcus* spp.^53,54^. Furthermore, Stalder *et al*.^55^ found that the presence of the *int1* gene increased the risk of the spread and transmission of resistance genes to environment and other bacteria.

Our findings, one *E*. *coli* isolate was detected both of the *mcr-3* and *int1* genes. This result corresponded with a previous study^49^ which had 24 out of 37 samples positive for both *int1* and *mcr* genes, and four samples were only positive for the *int1* gene.

The results of the antimicrobial effect of CFS from selected probiotics exhibited a decreased diameter of the inhibition zone as the incubation time continued. To support this finding, our results are consistent with previous studies^32,34,56^. In addition, Kaewchomphunuch *et al*.^32^ reported the CFS from *L*. *acidophilus*, *L*. *plantarum*, and *P*. *pentosaceus* which expressed inhibitory activity only to pathogenic *E*. *coli* isolated from pigs. Nevertheless, the previous study did not undertake a comprehensive study of the inhibitory activity against other pathogens or specific *E*. *coli* strain isolated from semen origin.

The CFS produced from *L*. *acidophilus* could inhibit the growth of *P*. *aeruginosa* while another activity was able to disrupt biofilms from *P*. *aeruginosa*^56^. The difference between El-Mokhtar’s study and the present study are the probiotics for CFS collection. Our study collected from other probiotics in lactic acid bacteria (LAB) including *P*. *acidilactici*, *P*. *pentosaceus*, and *L*. *plantarum* which showed similar results.

From the results of the present study, *P*. *mirabilis* could be inhibited by CFS from selected probiotics except for *B*. *axarquiensis* and *B*. *subtilis*. The inhibitory results are similar to Shaaban *et al*.^57^ study which mentioned the action from *L*. *casei* and *L*. *reuteri*. Besides, the inhibitory activities of pathogen growth inhibition derived from the CFS of *L*. *casei* and *L*. *reuteri* were able to inhibit *P*. *mirabilis* biofilm formation as well.

Factors that affect the ability to inhibit pathogens that depend on pH or concentration of antimicrobial compound including lactic and acetic acid or antimicrobial peptides (AMPs)^57,58^. The comparable findings of CFS from *L*. *johnsonii* was between pH 3.5 and pH 6.0. In addition, it has been reported that lower pH (acidic condition) could inhibit growth of *B*. *cereus* while higher pH causes the activity to disappear^58^. The reduced antimicrobial activity of CFS from *Lactobacillus* spp. and *Enterococcus* spp. cultures were found when their pH value was greater than 4.5^59^. However, a study of CFS from *L*. *plantarum* adjusted the pH up to 6.5 and the antimicrobial activity was still maintained^34^. In the same direction, Soria and Audisio^58^ revealed that the different compound in CFS also influenced the inhibitory activity of bacterial growth. From the present results, the pH value was observed as a main feature in the antimicrobial activity of CFS, the key compound in CFS for inhibiting pathogen growth might be that of AMPs and/or bacteriocins. With regard to the pH influence on inhibitory activity, our 3 CFSs produced from *Lactobacillus plantarum* NN31-5B, *Pediococcus acidilactici* NN82-7M and *Pediococcus pentosaceus* NN115-6M were neutralized to pH 6.0 and performed the antimicrobial activities with boar semen pathogens by agar well diffusion assay (data not shown). The inhibition zone was decreased but still inhibit the pathogens which showed similar results to other studies^34,56,57^. This can be suggested that our CFS might be contained antimicrobial peptides and/or bacteriocin with their antimicrobial properties. The AMPs were isolated from CFS (subtilosin) derived from *B*. *amyloliquefaciens* and could inhibit the bacterial vaginosis associated bacteria similar with the subtilosin from *B*. *subtilis*^60^. In addition, organic acid was found to be an essential compound of CFS derived from LAB. Nevertheless, Arrioja-Bretón *et al*.^34^ mentioned that the acid was not the only factor inhibiting bacterial growth. To support this hypothesis, Tenea^61^ demonstrated that the AMPs extracted from LAB-producing CFS (*L*. *plantarum* and *Lactococcus lactis*) could also inhibit against *Salmonella enterica*.

Although the previous studies were conducted to investigate the inhibitory effect of CFS derived from probiotics with some food-poisoning bacteria, our recent study successfully declares the inhibitory effect against pathogenic bacteria carrying antimicrobial resistance genes isolated from boar semen. However, further studies are needed to identify the constituent antimicrobial compound in this CFS, construct and synthetic these AMPs. Furthermore, the interaction between synthetic antimicrobial peptides and spermatozoa and the synthetic antimicrobial peptides-based semen extender without antibiotics will be included in a further study to determine its effect on the qualities of spermatozoa and field fertility.

## Conclusions

In conclusion, using bacterial contaminated fresh boar semen for artificial insemination is a possible cause of sow endometritis. Consequently, antibiotics are added to the boar semen extender for artificial insemination. Fortunately, CFS derived from probiotics can effectively inhibit bacteria carrying antimicrobial resistance genes isolated from fresh boar semen, in particular the CFS produced from the LAB. However, the ability against bacteria is observed and decreased with more extended incubation periods. In the present results, CFS with its antimicrobial peptides and/or bacteriocin constituent inhibits semen-origin pathogenic bacteria provide the direction as a promising alternative antibiotics method used in the semen extender preservation of the pig industry.

## Materials and methods

### Sample collection

Total semen samples (n = 10) were collected from 10 individual boars in three distinct AI centers in commercial pig farms in Thailand. Boars were housed in an individual pen in an evaporative cooling house system. They were fed with a commercial feed 3 kg once a day and the water were ad libitum. The sampling locations were conducted in Chai Nat province (farm A; n = 5), Chon Buri province (farm B; n = 3), and Chachoengsao province (farm C; n = 2). The boar semen samples were collected using the gloved hand technique and the whole ejaculate was filtrated with sterile gauze to eliminate the gel-rich fraction^7^. Then, only a fresh sperm-rich fraction was stored in sterile container. All specimens were preserved under the sterile repository at 4 °C and immediately shipped to the Laboratory of Bacteria, Veterinary Diagnostic Center, Faculty of Veterinary Science, Mahidol University. The research ethics was approved by the Faculty of Veterinary Science, Mahidol University-Institute Animal Care and Use Committee (FVS-MU-IACUC-Protocol No. MUVS-2021-10-41), Animal use license No. U1-01281-2558. All methods were performed in accordance with the relevant guidelines and regulations.

### Bacterial isolation and species identification

All semen samples were cultured on tryptone soy agar (Oxoid, UK) with 5% sheep blood and MacConkey agar (Oxoid, UK) incubated at 37 °C for 18-24 h. All different colonies were identified using standard biochemical tests followed by 16S rRNA sequencing and stored in Brain Heart Infusion (BHI) (Oxoid, UK) with 20% glycerol at -80 °C. Genomic DNA of all isolates was performed using G-spin^TM^ genomic DNA extraction kit (iNtRON, Republic of Korea) and amplified 16S rRNA by PCR with a BiometraTOne96G thermal cycler (AnalytikJena, Germany) using UFUL (5’- GCCTAACACATGCAAGTCGA-3’) and 800R (5’-TACCAGGGTATCTAATCC-3’) primers. The PCR was performed with the following protocol: initial denaturation at 94 °C for 3 min followed by 30 cycles of denaturation at 94 °C for 30 sec, annealing at 55 °C for 30 sec, and extension at 72 °C for 45 sec, with a final extension step at 72 °C for 5 min. The PCR products were purified by MEGAquick-spin^TM^ Plus Total Fragment DNA purification kit (iNtRON, Republic of Korea) and sequenced with an Applied Biosystems 3730XL DNA Analyzer (Bionics, Republic of Korea). Each 16S rRNA sequences was blasted against the NCBI nucleotide database (https://blast.ncbi.nlm.nih.gov) to identify all isolates.

### Antimicrobial susceptibility testing (AST)

All isolates were streaked onto blood agar to obtain single colony. Following incubation at 37 °C for 18-24 h, one to three colonies with similar morphological appearance were transferred into normal saline solution (0.85% NaCl) and thoroughly mixed. The turbidity of bacterial suspension was measured using 0.5 McFarland standard (approximately 10^8^ CFU/mL). The minimum inhibitory concentrations (MIC) were conducted by the broth microdilution with following a guideline from the Clinical and Laboratory Standards Institute (CLSI). The assays were performed in triplicate with 96 well plates; in each well, 100 μL of bacterial suspension previously diluted in Mueller Hinton broth (Difco, USA) to 10^6^ CFU/mL were added to 100 μL of appropriate dilutions of antimicrobials. A total of 10 antimicrobials were tested in the following concentrations by means of two-fold dilution: amoxicillin (TCI, Japan) 1-128 μg/mL, amoxicillin trihydrate:potassium clavulanate (4:1; Sigma, Germany) 1-128 μg/mL, ceftazidime (Sigma, Germany) 1-128 μg/mL, ceftriaxone (TCI, Japan) 0.25-32 μg/mL, ceftiofur (TCI,Japan) 0.25-32 μg/mL, colistin (Sigma, Germany) 0.25-32 μg/mL, enrofloxacin (Fluka Biochemika, Japan) 0.06-8 μg/mL, gentamicin (TCI, Japan) 0.5-64 μg/mL, oxytetracycline (AppliChem, USA) 0.25-32 μg/mL, and trimethoprim:sulfamethoxazole (1:19; TCI, Japan) 0.25-32 μg/mL. The 96 well plates were incubated at 37 °C for 16-20 h. Medium without antimicrobials was conducted as control and inoculated prior to and following each antimicrobial-containing series of plates. MIC values were recorded after incubation and defined as the lowest concentration of each antibiotic without a visible growth of bacteria. The reference strains *Escherichia coli* ATCC 25922 and *Pseudomonas aeruginosa* ATCC 27853 were conducted in each experiment to assess the reliability of methodology.

### Detection of *mcr* and* int1* genes

Bacterial plasmid DNA was extracted from all pathogens by using QIAprep Spin Miniprep Kit (Qiagen, Germany) with following the manufacturer’s instruction. DNA concentrations were measured using a BioDrop DUO (DKSH, UK). The plasmid-mediated colistin resistance genes (*mcr-1* to *mcr-10*) and the class 1 integron-integrase gene (*int1*) were detected by multiplex PCR using Green PCR master mix kit (Biotechrabbit, Germany) with following the Nguyet *et al*. protocol^49^ of the primers and PCR conditions. Briefly, the amplification steps were performed using BiometraTOne96G (AnalytikJena, Germany) with the following thermal cycles: the initial denaturation at 94 °C for 3 min, followed by 25 cycles denaturation at 94 °C for 30 sec, annealing at 58 °C for 90 sec, and extension at 72 °C for 60 sec, a final extension step at 72 °C for 5 min. The PCR products were separated using 1.5% agarose gel electrophoresis, stained with 1X GelRed (Sigma Aldrich, USA), and visualized under an UV transilluminator UVP GelStudio (AnalytikJena, USA). The reference strain *E*. *coli* ATCC 25922 was conducted as a negative control strain, while *E*. *coli* harboring *mcr* genes were conducted as a positive control strain.

### Probiotic strains used

The probiotic strains used in our study were thoroughly considered and then selected the probiotics which were supported and based on our preliminary and previous studies. To exemplify, Kaewchomphunuch *et al*.^32^ reported on the inhibitory activities of *Lactobacillus* spp., *Pediococcus* spp., and *Enterococcus* spp. against pathogenic *E*. *coli* isolated from pigs in Thailand^32^. Aupad *et al*.^36^ also carried out the antibacterial activities of isolated *Bacillus* spp. against bacteria found in food. The multidrug-resistant *E*. *coli* subjected to the *Weissella confusa* activity were investigated by Dey *et al*.^37^. Thus, the probiotic candidates were listed in Table 2.

### Cell free supernatants (CFS) preparation from selected probiotics

The probiotic strains used in this study were obtained from a collection of bacterial stock cultures which stored in glycerol at -80 °C from the Laboratory of Bacteria, Veterinary Diagnostic Center, Faculty of Veterinary Science, Mahidol University. The CFS was prepared according to the Kaewchomphunuch *et al*. protocol^32^. Briefly, overnight MRS culture broth of eight selected probiotics (Table 2) was transferred into 1.5 mL microcentrifuge tube and centrifuged for 2 min at 5,000 rpm (Denville Micro 260D Microcentrifuge, Denville Scientific, Inc., Metuchen, USA). Supernatants were collected by pass through 0.22 μm sterile syringe filter (Guangzhou Jet Bio-Filtration Co., Ltd., Guangzhou, China). The filtrated CFS was either used freshly in agar well diffusion assay or stored at -20 °C for further analysis.

### Agar well diffusion assay

*Pseudomonas aeruginosa*, *E*. *coli*, and *Proteus mirabilis* isolated from boar semen were subjected for testing the inhibitory effect of CFS from probiotics. All bacteria were cultured in BHI broth at 37 °C for 20-24 h. Bacterial suspension was initially diluted into to 0.5 McFarland standard and performed spread plate method onto nutrient agar. Then, the inoculated nutrient agar was pierced with the sterile 8 mm diameter cork borer to create wells. The volume of 100 μL of CFS was loaded into wells and incubated at 37 °C for 8, 10, 12, 14, and 16 h. After incubation, the inhibition zone will be measured in each well. To validate the result, MRS broth (pH 6.0) was conducted as a negative control.

### Statistical analysis

The descriptive statistic was used in this study. In addition, the data analysis was performed by using one-way analysis of variance (ANOVA) and compared means by using Duncan’s test by The PASW Statistics for Windows, version 18.0 (SPSS Inc., Chicago, IL, USA). A statistical significance is determined as *p* value < 0.05.

### Ethics declarations

The study was conducted in compliance with the ARRIVE guidelines. The research ethics was approved by the Faculty of Veterinary Science, Mahidol University-Institute Animal Care and Use Committee (FVS-MU-IACUC-Protocol No. MUVS-2021-10-41), Animal use license No. U1-01281-2558.

## Data Availability

The datasets generated and/or analysed during the current study are available in the NCBI GenBank database under the accession numbers OQ626730, OQ626774, OQ626814, OQ626831, OQ626832, OQ626905, OQ627018, OQ627030, OQ627212, OQ627311, OQ627369, OQ627374, OQ627392, OQ627394, OQ627406, OQ627411, OQ627412, OQ627431, and OQ627435.

## Abbreviations

AI: Artificial insemination
AMPs: Antimicrobial peptides
AMR: Antimicrobial resistance
AST: Antibiotic susceptibility test
BHI: Brain heart infusion medium
CFS: Cell free supernatants
*int1*: Class 1 integron-integrase
CLSI: Clinical and laboratory standards institute
LAB: Lactic acid bacteria
LPS: Lipopolysaccharides
*mcr*: Mobile colistin resistance
MIC: Minimum inhibitory concentrations
MRS: De man, rogosa, and sharpe medium
PCR: Polymerase chain reaction
PEA: Phosphoethanolamine

## References

[1] Maes D (2008). Diseases in swine transmitted by artificial insemination: An overview. Theriogenology.

[2] Kuster CE, Althouse GC (2016). The impact of bacteriospermia on boar sperm storage and reproductive performance. Theriogenology.

[3] Farnum, D. W. & Riese, R. L. Urogenital infections in sows and gilts; differential diagnosis, diagnostic techniques and control. *Iowa State Univ*. *Vet*. **51**, 1–5. https://core.ac.uk/reader/38906280 (1989).

[4] de Winter P, Verdoncka M, de Kruif A, Devriese L, Haesebrouck F (1995). Bacterial endometritis and vaginal discharge in the sow: Prevalence of different bacterial species and experimental reproduction of the syndrome. Anim. Reprod. Sci..

[5] Lang A, Kaeoket K, Kindahl H, Madej A, Einarsson S (2004). Influence of CRH and ACTH administration on endocrine profile and ovulation in sows. Reprod. Domest. Anim..

[6] Kaeoket K, Persson E, Dalin AM (2003). Influence of pre-ovulatory insemination and early pregnancy on the distribution of CD2, CD4, CD8 and MHC class II expressing cells in the sow endometrium. Anim. Reprod. Sci..

[7] Kaeoket K, Tantasuparuk W, Kunavongkrit A (2005). The effect of post-ovulatory insemination on the subsequent embryonic loss, oestrous cycle length and vaginal discharge in sows. Reprod. Domest. Anim..

[8] Pezo F, Romero F, Zambrano F, Sánchez RS (2019). Preservation of boar semen: An update. Reprod. Domest. Anim..

[9] Althouse GC, Lu KG (2005). Bacteriospermia in extended porcine semen. Theriogenology.

[10] Gączarzewicz D, Udała J, Piasecka M, Błaszczyk B, Stankiewicz T (2016). Bacterial contamination of boar semen and its relationship to sperm quality preserved in commercial extender containing gentamicin sulfate. Pol. J. Vet. Sci..

[11] Okazaki T (2010). Polymyxin B neutralizes bacteria-released endotoxin and improves the quality of boar sperm during liquid storage and cryopreservation. Theriogenology.

[12] Maroto Martín LO (2010). Bacterial contamination of boar semen affects the litter size. Anim. Reprod. Sci..

[13] Schulze M, Dathe M, Waberski D, Müller K (2016). Liquid storage of boar semen: Current and future perspectives on the use of cationic antimicrobial peptides to replace antibiotics in semen extenders. Theriogenology.

[14] Schulze M (2017). Dose rates of antimicrobial substances in boar semen preservation—time to establish new protocols. Reprod. Domest. Anim..

[15] Vickram A, Ramesh Pathy M, Sridharan T (2017). Preputial washing, addition of antioxidants and antimicrobial peptides in semen extender- for reducing microbial load during cryopreservation. JSM Invitro. Fertil..

[16] Santos, C. S. & Silva, A. R. Current and alternative trends in antibacterial agents used in mammalian semen technology. *Anim*. *Reprod*. **17**, e20190111. 10.21451/1984-3143-AR2019-0111 (2020). 10.21451/1984-3143-AR2019-0111 PMC7212743 32399069

[17] Morrell JM, Wallgren M (2014). Alternatives to antibiotics in semen extenders: A review. Pathogens.

[18] Gadea J (2003). Review: Semen extenders used in the artificial insemination of swine. Span. J. Agric. Res..

[19] Bryła M, Trzcińska M (2015). Quality and fertilizing capacity of boar spermatozoa during liquid storage in extender supplemented with different antibiotics. Anim. Reprod. Sci..

[20] Morrell J (2016). Antimicrobials in boar semen extenders—A risk/benefit analysis. J. Antimicrob. Agents.

[21] Koyama Y (1950). A new antibiotic “colistin” produced by spore-forming soil bacteria. J. Antibiot..

[22] El-Sayed Ahmed MAEG (2020). Colistin and its role in the era of antibiotic resistance: An extended review (2000–2019). Emerg. Microbes. Infect..

[23] Hussein, N. H., AL-Kadmy, I. M. S., Taha, B. M. & Hussein, J. D. Mobilized colistin resistance (mcr) genes from 1 to 10: A comprehensive review. Mol. Biol. Rep. **48**, 2897–2907. 10.1007/s11033-021-06307-y (2021). 10.1007/s11033-021-06307-y 33839987

[24] Carroll LM (2019). Identification of novel mobilized colistin resistance gene *mcr-9* in a multidrug-resistant, colistin-susceptible *Salmonella enterica* serotype Typhimurium isolate. mBio.

[25] Xu Y, Lin J, Cui T, Srinivas S, Feng Y (2018). Mechanistic insights into transferable polymyxin resistance among gut bacteria. J. Biol. Chem..

[26] Lv J (2018). Discovery of a *mcr-1*-bearing plasmid in commensal colistin-resistant *Escherichia coli* from healthy broilers in Faisalabad. Pakistan. Virulence.

[27] Sun J (2017). Deciphering *mcr-2* colistin resistance. mBio.

[28] Akrami, F., Rajabnia, M. & Pournajaf, A Resistance integrons: A mini review. Caspian J. Intern. Med. **10**, 370–376. 10.22088/cjim.10.4.370, (2019). 10.22088/cjim.10.4.370 PMC6856922 31814933

[29] Baltazar, M. *et al.* Activation of class 1 integron integrase is promoted in the intestinal environment. PLoS Genet. **18**, e1010177. 10.1371/journal.pgen.1010177 (2022). 10.1371/journal.pgen.1010177 PMC9090394 35482826

[30] Boucher Y, Labbate M, Koenig JE, Stokes HW (2007). Integrons: Mobilizable platforms that promote genetic diversity in bacteria. Trends Microbiol..

[31] Tenea GN, Barrigas A (2018). The efficacy of bacteriocin-containing cell-free supernatant from *Lactobacillus plantarum* Cys5-4 to control pathogenic bacteria growth in artisanal beverages. Int. Food. Res. J..

[32] Kaewchomphunuch, T., Charoenpichitnunt, T., Thongbaiyai, V., Ngamwongsatit, N. & Kaeoket, K. Cell-free culture supernatants of Lactobacillus spp. and Pediococcus spp. inhibit growth of pathogenic Escherichia coli isolated from pigs in Thailand. BMC Vet. Res. **18**, 60. 10.1186/s12917-022-03140-8 (2022). 10.1186/s12917-022-03140-8 PMC8800250 35093088

[33] Pelyuntha W, Chaiyasut C, Kantachote D, Sirilun S (2019). Cell-free supernatants from cultures of lactic acid bacteria isolated from fermented grape as biocontrol against *Salmonella* Typhi and *Salmonella* Typhimurium virulence via autoinducer-2 and biofilm interference. PeerJ.

[34] Arrioja-Bretón D, Mani-López E, Bach H, López-Malo A (2020). Antimicrobial activity of protein-containing fractions isolated from *Lactobacillus plantarum* NRRL B-4496 culture. Braz. J. Microbiol..

[35] Hartmann HA, Wilke T, Erdmann R (2011). Efficacy of bacteriocin-containing cell-free culture supernatants from lactic acid bacteria to control *Listeria monocytogenes* in food. Int. J. Food Microbiol..

[36] Aupad R, Sripotong N, Khamlak K, Inchidjuy S, Rattanasinganchan P, Pipatsatitpong D (2011). Isolation and characterization of bacteriocin with anti-listeria and anti-MRSA activity produced by food and soil isolated bacteria. Afr. J. Microbial. Res..

[37] Dey DK, Khan I, Kang SC (2019). Anti-bacterial susceptibility profiling of *Weissella confusa* DD_A7 against the multidrug-resistant ESBL-positive. E. Coli. Microd. Pathog..

[38] Dalmutt AC (2020). Characterization of bacterial contaminants of boar semen: Identification by MALDI-TOF mass spectrometry and antimicrobial susceptibility profiling. J. Appl. Anim. Res..

[39] Wang Y (2020). Isolation and characteristics of multi-drug resistant *Streptococcus porcinus* from the vaginal secretions of sow with endometritis. BMC Vet. Res..

[40] Burch DGS, Sperling D (2018). Amoxicillin—current use in swine medicine. J. Vet. Pharmacol. Ther..

[41] Bresciani C (2014). Boar semen bacterial contamination in Italy and antibiotic efficacy in a modified extender. Ital. J. Anim. Sci..

[42] Costinar L (2021). Boar semen contamination: Identification of Gram-negative bacteria and antimicrobial resistance profile. Animals.

[43] Morrell JM (2019). Removal of bacteria from boar semen using a low-density colloid. Theriogenology.

[44] Hensel B (2020). Low temperature preservation of porcine semen: Influence of short antimicrobial lipopeptides on sperm quality and bacterial load. Sci. Rep..

[45] Shaoyong W (2019). Effects of kojic acid on boar sperm quality and anti-bacterial activity during liquid preservation at 17 C. Theriogenology.

[46] Schulze M (2019). Antibacterial defense and sperm quality in boar ejaculates. J. Reprod. Immunol..

[47] Jäkel H (2021). *In vitro* performance and *in vivo* fertility of antibiotic-free preserved boar semen stored at 5 °C. J. Anim. Sci. Biotechnol..

[48] García V (2018). Co-occurrence of *mcr-1*, *mcr-4* and *mcr-5* genes in multidrug-resistant ST10 Enterotoxigenic and Shiga toxin-producing *Escherichia coli* in Spain (2006–2017). Int. J. Antimicrob. Agents.

[49] Nguyet LTY, Keeratikunakorn K, Kaeoket K, Ngamwongsatit N (2022). Antibiotic resistant *Escherichia coli* from diarrheic piglets from pig farms in Thailand that harbor colistin-resistant *mcr* genes. Sci. Rep..

[50] Mmatli M, Mbelle NM, Osei Sekyere J (2022). Global epidemiology, genetic environment, risk factors and therapeutic prospects of *mcr* genes: A current and emerging update. Front. Cell. Infect. Microbiol..

[51] de La Torre E (2014). Detection of integrase gene in E. coli isolated from pigs at different stages of production system. Int. J. Microbiol..

[52] Deng Y (2015). Resistance integrons: Class 1, 2 and 3 integrons. Ann. Clin. Microbiol. Antimicrob..

[53] Veise, P., Ramazanzadeh, R., Khiababi, Z. D., Derakhshi, B. & Amirmozafari, N. Identification of class I integrons gene in staphylococcus strains isolated from clinical samples. Cell Biolo., **1**, 24–27. 10.11648/j.cb.20130103.11, (2013)

[54] Ye C (2020). Prevalence and characterisation of class 1 and 2 integrons in multi-drug resistant *Staphylococcus aureus* isolates from pig farms in Chongqing. China. J. Vet. Res..

[55] Stalder T, Barraud O, Casellas M, Dagot C, Ploy MC (2012). Integron involvement in environmental spread of antibiotic resistance. Front. Microbiol..

[56] El-Mokhtar MA (2020). Antagonistic activities of cell-free supernatants of *Lactobacilli* against extended-spectrum β-lactamase producing *Klebsiella pneumoniae* and *Pseudomonas aeruginosa*. Infect. Drug. Resist..

[57] Shaaban M, El-Rahman OAA, Al-Qaidi B, Ashour HM (2020). Antimicrobial and antibiofilm activities of probiotic *Lactobacilli* on antibiotic-resistant *Proteus mirabilis*. Microorganisms.

[58] Soria MC, Audisio MC (2014). Inhibition of *Bacillus cereus* strains by antimicrobial metabolites from *Lactobacillus johnsonii* CRL1647 and *Enterococcus faecium* SM21. Prob. Antimicrob. Prot..

[59] Kralik P, Babak V, Dziedzinska R (2018). The impact of the antimicrobial compounds produced by lactic acid bacteria on the growth performance of Mycobacterium avium subsp. paratuberculosis. Front. Microbiol..

[60] Sutyak KE, Wirawan RE, Aroutcheva AA, Chikindas ML (2008). Isolation of the *Bacillus subtilis* antimicrobial peptide subtilosin from the dairy product-derived *Bacillus amyloliquefaciens*. J. Appl. Microbiol..

[61] Tenea GN (2020). Peptide extracts from native lactic acid bacteria generate ghost cells and spheroplasts upon interaction with *Salmonella enterica*, as promising food antimicrobials. BioMed. Res. Int..

